# YAP Acts as a Negative Regulator of Mini Utrophin-Based Gene Therapy for Duchenne Muscular Dystrophy in Mdx Mice

**DOI:** 10.3390/ijms27094108

**Published:** 2026-05-04

**Authors:** Zhuo Li, Yafeng Song

**Affiliations:** 1 School of Sports Science, Beijing Sport University, No. 48, Xinxi Road, Haidian District, Beijing 100084, China; 2 China Institute of Sport and Health Science, Beijing Sport University, No. 48, Xinxi Road, Haidian District, Beijing 100084, China

**Keywords:** Duchenne muscular dystrophy, gene therapy, MyoAAV, mini utrophin, YAP

## Abstract

Duchenne muscular dystrophy (DMD) is a fatal rare disease caused by dystrophin deficiency, with no effective clinical treatments available to date. Using mdx mice as a model, this study investigated the therapeutic efficacy and interaction of mini utrophin (a truncated utrophin) and Yes-associated protein (YAP) delivered via recombinant adeno-associated virus (rAAV). Results showed that mini utrophin was efficiently expressed in mdx mouse skeletal muscle, significantly increased phosphorylated YAP (p-YAP) levels, restored the expression of dystrophin–glycoprotein complex (DGC) components (α/γ-sarcoglycans), reduced serum creatine kinase (CK) leakage, alleviated pathological damages such as central nucleation and inflammatory infiltration, and comprehensively improved grip strength, treadmill endurance, and pole climbing ability in mice. However, the co-overexpression of YAP completely antagonized these therapeutic effects, resulting in no improvement in pathological phenotypes or motor function of mdx mice. This study confirms that mini utrophin can effectively reverse DMD-related phenotypes, while excessive YAP activation abrogates its therapeutic efficacy, suggesting that precise regulation of YAP activity is required in DMD treatment and providing experimental basis for optimizing gene therapy strategies.

## 1. Introduction

Duchenne muscular dystrophy (DMD) is an X-linked recessive neuromuscular disorder caused by mutations in the DMD gene. The incidence of DMD is approximately 1 in 3500–5000 live male births. The disease is characterised by progressive skeletal muscle fibre necrosis, inflammation, and motor dysfunction due to the loss of dystrophin, which is essential for maintaining sarcolemmal integrity and mechanotransduction [1]. Due to dystrophin deficiency, DMD is accompanied by a cascade of pathological events in skeletal muscle, including disrupted ion homeostasis of the sarcolemma, overactivation of calpains, and impaired mitochondrial function [2,3]. These findings fully demonstrate that DMD, caused by DMD gene mutations, is a severe neuromuscular disorder associated with complex impairments of physiological functions. As indicated in our previous research, utrophin, a dystrophin homologue, has been identified as a highly promising therapeutic target for DMD [4,5].

Yes-associated protein (YAP), which is widely expressed in various tissues and cells, has been demonstrated to promote cell proliferation, inhibit excessive differentiation, and maintain regenerative potential in cardiomyocytes and skeletal muscle satellite cells [6,7]. Emerging evidence also highlights the role of YAP as a central mediator of mechanotransduction in skeletal muscle [8,9]. YAP is anchored to the sarcolemma via binding to β-dystroglycan of the DGC, and its activity is tightly regulated by mechanical cues [10]. The hypothesis of this study is that upregulating the expression of the transcription factor YAP in conjunction with the utrophin-mediated restoration of the pathological structure of skeletal muscle would result in a synergistic enhancement of skeletal muscle function in mdx mice, a classic DMD animal model. 

In this study, the therapeutic effects of mini utrophin and Yap, delivered via recombinant adeno-associated virus (rAAV), on motor function and muscle pathology in mdx mice were evaluated.

## 2. Results

### 2.1. Successful Delivery of Mini Utrophin and Yap in Mdx Mice Without Yap-Mediated Amelioration of Muscle Pathology

**Figure 1:**
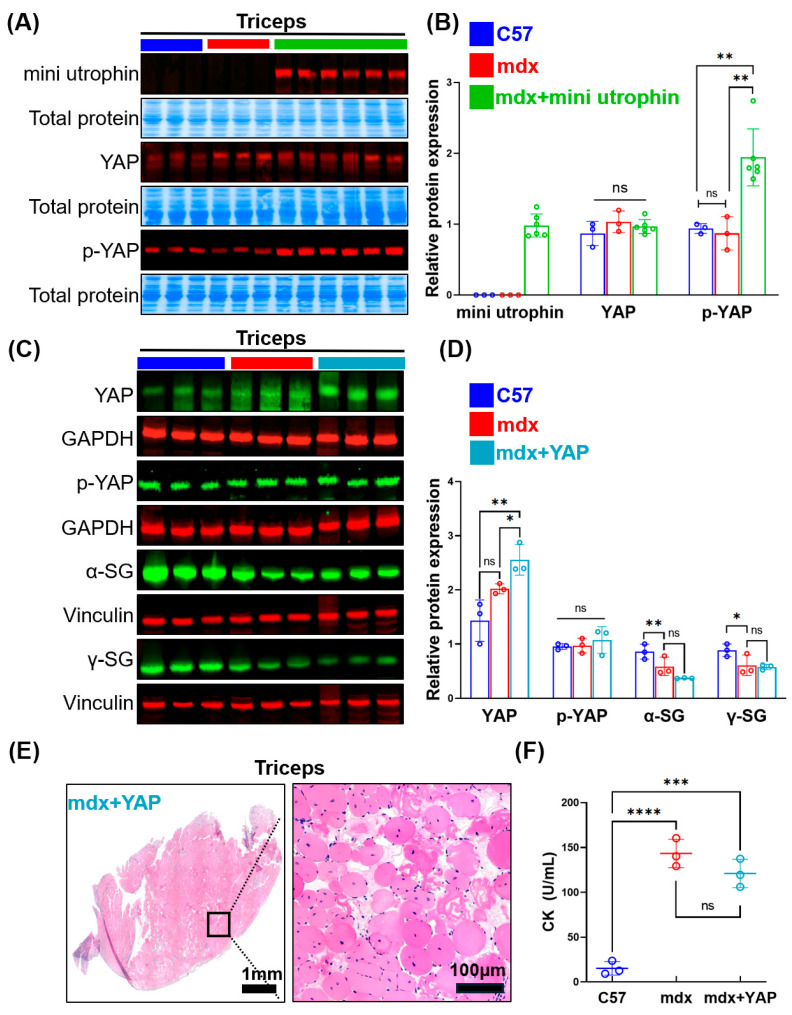
Successful expression of exogenous mini utrophin in skeletal muscle of mdx mice: (**A**) Representative pictures of Western blotting indicating the protein levels of the mini utrophin (~112 kDa), YAP (~65 kDa), and p-YAP (~65 kDa) in triceps tissues; (**B**) Quantitative analysis of protein levels (*n* = 3 in C57 and mdx groups, *n* = 6 in mdx + mini utrophin group); (**C**) Representative pictures of Western blotting indicating the YAP (~65 kDa), p-YAP (~65 kDa), α-Sarcoglycan (~50 kDa), γ-Sarcoglycan (~35 kDa), GAPDH (~37 kDa), and Vinculin(~114 kDa) in triceps tissues; (**D**) Quantitative analysis of YAP levels (*n* = 3/group); (**E**) HE staining of the triceps in the mdx + YAP group; (**F**) Quantified levels of creatine kinase from mice serum samples (*n* = 3/group) assessed by creatine kinase activity assay kit. Symbol ns indicates no significant difference, * indicates *p* ≤ 0.05, ** indicates *p* ≤ 0.01, *** indicates *p* ≤ 0.001, and **** indicates *p* ≤ 0.0001 between the relevant groups that are compared.

No mini utrophin expression was detected in the skeletal muscle of C57 and mdx groups, whereas robust mini utrophin expression was achieved in the mdx + mini utrophin group (Figure 1A). YAP expression showed no significant difference among the three groups; notably, compared with the C57 and mdx groups, the mdx + mini utrophin group exhibited a marked increase in p-YAP expression (*p* ≤ 0.01) (Figure 1A,B). MyoAAV was used to deliver YAP to mdx mice. Western blotting results showed that YAP was overexpressed and activated in the mdx + YAP group, whereas phosphorylated YAP (p-YAP) was not significantly upregulated with increased YAP levels. Compared with the mdx group, there were no significant differences in the expression of dystrophin–glycoprotein complex (DGC) components in the mdx + YAP group (Figure 1C,D). HE staining and serum creatine kinase (CK) assays showed no difference in pathological features between the mdx + YAP and mdx groups (Figure 1E,F). These findings confirm efficient rAAV-mediated infection of skeletal muscle and expression of mini utrophin and YAP, and demonstrate that mini utrophin delivery induces significant phosphorylation of YAP, suggesting a potential functional association between mini utrophin-mediated DGC restoration and YAP signalling. However, YAP delivery alone did not ameliorate the typical pathological features of mdx mice.

### 2.2. Antagonistic Roles of Mini Utrophin and YAP in DGC Complex Expression

**Figure 2:**
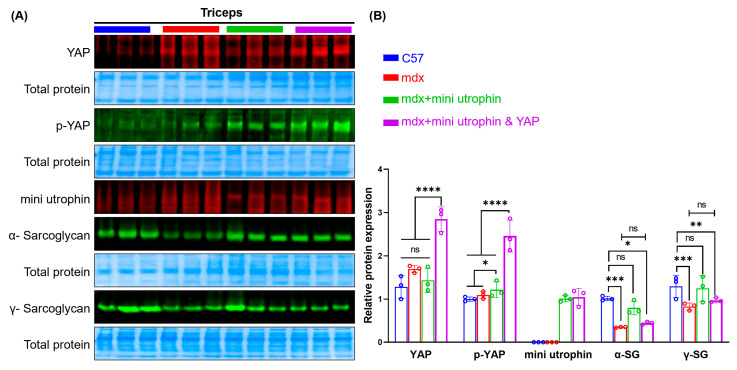
Expression of the related protein in the triceps and diaphragm of the mdx mice. (**A**) Representative pictures of Western blotting indicating the protein levels of the mini utrophin (~112 kDa), YAP (~65 kDa), p-YAP (~65 kDa), α-Sarcoglycan (~50 kDa), and γ-Sarcoglycan (~35 kDa) in triceps; (**B**) Quantitative analysis of protein levels in triceps (*n* = 3/group). Symbol ns indicates no significant difference, * indicates *p* ≤ 0.05, ** indicates *p* ≤ 0.01, *** indicates *p* ≤ 0.001, and **** indicates *p* ≤ 0.0001.

The mdx model received a co-injection of rAAVs encoding mini utrophin and YAP, and protein levels in the triceps brachii were measured. Mini utrophin was undetectable in the C57 and mdx groups but expressed in the mdx + mini utrophin group. YAP and *p*-YAP levels were markedly elevated in the mdx + mini utrophin and YAP group compared to other groups. αSG and γSG expression did not differ between mdx + mini utrophin and YAP and mdx groups, nor between mdx + mini utrophin and C57 groups (Figure 2). This suggests mini utrophin alone improves DGC expression in mdx mice, while the co-overexpression of YAP counteracts its DGC-recruiting effect.

### 2.3. Mini Utrophin Ameliorates Skeletal Muscle Pathological Phenotypes in Mdx Mice and the Antagonistic Effect of YAP

**Figure 3:**
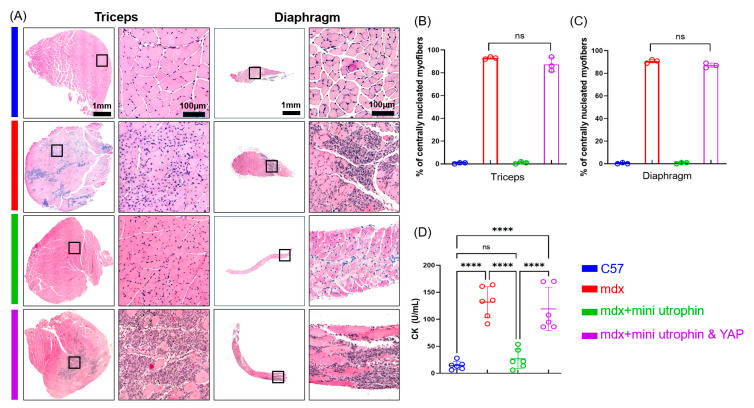
Mini utrophin ameliorates dystrophic pathology in skeletal muscle of mdx mice, whereas YAP demonstrates no therapeutic efficacy. (**A**) HE-stained images of different groups. (**B**,**C**) Bar graph showing quantified central nuclear myofibrils (*n* = 3/group). (**D**) Quantified levels of creatine kinase from mice serum samples (*n* = 6/group) assessed by creatine kinase activity assay kit. Symbol ns indicates no significant difference; **** indicates *p* ≤ 0.0001.

Hematoxylin–Eosin staining showed typical DMD pathology (central nuclei, inflammatory infiltration) in mdx mouse skeletal muscle. These pathological changes were obviously abrogated in the mdx + mini utrophin group across different skeletal muscle regions (Figure 3A–C). However, the mdx + mini utrophin and YAP group displayed typical DMD phenotypes, identical to the mdx control. Serum CK levels were measured at the experimental endpoint. Compared with C57 controls, mdx mice had significantly higher CK levels (*p* ≤ 0.0001), while mdx + mini utrophin mice showed normalized CK levels. Notably, serum CK levels in the mdx + mini utrophin and YAP group differed significantly from the C57 group (*p* ≤ 0.0001) (Figure 3D). This demonstrates that mini utrophin alone improves mdx pathology, but co-overexpression with YAP fails to exert a therapeutic effect.

### 2.4. Effects of Combined Mini Utrophin and YAP Treatment on Motor Function in Mdx Mice

**Figure 4:**
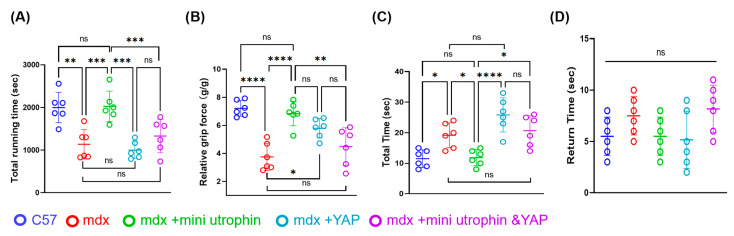
Differential effects of mini utrophin and YAP on behavioural performance in mdx mice. (**A**) Scatter plot of statistical results for mouse running time to exhaustion (*n* = 6/group). (**B**) Test results for relative grip strength of mouse limbs (*n* = 6/group). (**C**) Total duration results of the mouse pole climbing test (*n* = 6/group). (**D**) The scatter plot represents the results of the return time in the mice climbing pole test (*n* = 6/group). Symbol ns indicates no significant difference. Symbol ns indicates no significant difference, * indicates *p* ≤ 0.05, ** indicates *p* ≤ 0.01, *** indicates *p* ≤ 0.001, and **** indicates *p* ≤ 0.0001.

Behavioural tests were initiated at the end of week 5 following rAAV injection, including grip strength measurement, maximum treadmill endurance test, and pole climbing test. Results indicated that compared with the mdx group, the mdx + mini utrophin group exhibited significantly increased treadmill endurance time, grip strength, and pole climbing ability (Figure 4A–D), which were comparable to those of the C57 group. However, the mdx + YAP group and mdx + mini utrophin and YAP group still showed significant differences in motor function tests relative to the C57 group. These findings demonstrate that sole delivery of mini utrophin improves motor function in mdx mice, whereas the concurrent overexpression of mini utrophin and YAP fails to enhance their motor performance.

## 3. Discussion

Duchenne muscular dystrophy (DMD) is a devastating neuromuscular disorder driven by dystrophin deficiency and subsequent disruption of the dystrophin-glycoprotein complex (DGC), leading to sarcolemmal instability, muscle pathology, and functional impairment [11,12]. In this study, we demonstrate that systemic delivery of mini utrophin via rAAV (Myo2A-MHCK7-mini utrophin) effectively rescues DMD-related phenotypes in mdx mice, while concurrent YAP overexpression antagonizes these therapeutic benefits.

Previous studies have confirmed that utrophin, a dystrophin homologue, can restore the integrity of the dystrophin–glycoprotein complex (DGC), enhance motor function in mdx mice, and reverse skeletal muscle pathological features of DMD, making it a core candidate target for DMD gene therapy [4,13,14]. Our findings confirm that mini utrophin is successfully expressed in mdx mouse skeletal muscle, where it restores DGC integrity by normalizing α-sarcoglycan and γ-sarcoglycan levels. This structural repair translates to improved sarcolemmal stability, as evidenced by reduced serum CK leakage, amelioration of dystrophic histopathology (central nucleation and inflammatory infiltration), and significant enhancement of motor function (grip strength, treadmill endurance, and climbing performance) to wild-type levels. Additionally, mini utrophin treatment significantly upregulates phosphorylated YAP (p-YAP) expression, suggesting a potential regulatory crosstalk between utrophin-mediated DGC repair and YAP signalling. Importantly, our data do not demonstrate a direct physical interaction between mini utrophin and YAP. Rather, based on the known role of the DGC in mechanotransduction and YAP regulation, we speculate that mini utrophin may indirectly influence YAP activity by restoring membrane-associated structural integrity and the mechanical microenvironment of dystrophic muscle.

YAP is increasingly recognized as a central mechanotransduction effector that integrates changes in cytoskeletal tension, adhesion signaling, extracellular matrix stiffness, and Hippo pathway activity into transcriptional responses [8,15]. In skeletal muscle, YAP has been implicated in the regulation of myofiber size, regenerative signaling, and satellite cell fate, underscoring its context-dependent roles in both homeostasis and pathology [6,9,16]. In dystrophic muscle, where membrane fragility, fibrosis, altered mechanical load transmission, and repeated degeneration/regeneration cycles coexist, YAP-related signaling changes are likely to reflect complex mechanobiological adaptations rather than a single linear pathway alteration. Notably, co-administration of YAP-overexpressing rAAV abrogates all protective effects of mini utrophin. Mice receiving combined mini utrophin and YAP exhibit DGC component expression, histopathological features, serum CK levels, and motor function comparable to untreated mdx mice. This antagonistic effect underscores the critical importance of tightly regulated YAP activity in DMD pathogenesis. Aberrant exogenous YAP overexpression may breach this regulatory threshold, resulting in nuclear accumulation of overly active YAP. Such accumulation may negate the therapeutic benefits of mini utrophin through orchestrating downstream pro-inflammatory responses, excessive cell proliferation, and fibrogenic processes [17,18]. YAP signaling is subject to complex multilayered regulation, and neither total YAP abundance nor phosphorylation status alone is sufficient to define its functional activity. In canonical Hippo signaling, increased YAP phosphorylation is typically linked to cytoplasmic retention and reduced transcriptional output. Accordingly, interpretation of YAP signaling in dystrophic muscle requires an integrated assessment of protein abundance, phosphorylation, subcellular localization, and target gene activation. In this study, mini utrophin treatment and YAP overexpression were associated with alterations in YAP-related protein signals; however, in the absence of direct analyses of nuclear localization and YAP/TEAD-dependent transcription, these observations remain descriptive and correlative. It therefore remains to be determined whether the observed changes reflect modulation of YAP activity per se, compensatory signaling responses, or broader remodeling of mechanotransductive pathways. A limitation of this study is the lack of in-depth mechanistic research on the interaction between mini utrophin and YAP. In addition, the present work was performed only in mdx mice, in which endogenous utrophin is compensatorily upregulated due to dystrophin deficiency. This compensatory response may partially attenuate disease severity and could influence the observed therapeutic efficacy of exogenous mini utrophin. Therefore, the absence of validation in a more stringent model, such as mdx:utrophin double-knockout mice, limits the interpretation and generalizability of our findings. Future studies should assess mini utrophin and YAP interactions in severe dystrophic models lacking endogenous utrophin compensation, which will help clarify whether the antagonistic effect of YAP on mini utrophin therapy is preserved under these conditions and further define the utrophin-YAP-DGC regulatory axis. A further limitation is the absence of an empty AAV vector control. While this may be of limited impact in assessing the therapeutic effect of mini utrophin alone, it is more relevant for interpretation of the combined mini utrophin and YAP treatment groups, where potential vector-related or co-infection-related effects cannot be fully excluded. Accordingly, conclusions regarding their functional interaction should be considered with appropriate caution.

From a translational perspective, these findings are relevant to the broader development of DMD gene therapies. While micro- and mini dystrophin approaches have shown substantial promise, they remain constrained by vector cargo limitations, incomplete replacement of full-length dystrophin function, and possible immune responses to newly introduced dystrophin epitopes. As an endogenous dystrophin paralogue, utrophin offers a mutation-independent alternative with potentially lower immunogenicity. Our study therefore supports not only the therapeutic value of mini utrophin, but also the concept that intracellular signaling context may influence the effectiveness of utrophin-based therapy.

In summary, systemic mini utrophin delivery improved muscle pathology, restored DGC-associated proteins, and enhanced functional performance in mdx mice, supporting its therapeutic potential for DMD. Our data further suggest a potential association between mini utrophin-mediated rescue and YAP-related signaling changes, indicating a possible link between structural membrane repair and mechanotransduction-associated pathways in dystrophic muscle. However, because YAP activity was not directly assessed through analyses of nuclear localization, transcriptional activity, or downstream target gene expression, this relationship should be considered descriptive and correlative. Further mechanistic studies are needed to determine whether and how YAP-dependent signaling contributes to utrophin-based rescue.

## 4. Materials and Methods

### 4.1. Preparation of rAAV Vectors

Recombinant AAV (rAAV) vectors were constructed using the engineered muscle-tropic capsid MyoAAV 2A (Myo2A). MyoAAV 2A is an engineered AAV9-derived capsid optimized for efficient skeletal muscle transduction [19].

The transgene expression cassettes were flanked by AAV ITRs and driven by the muscle-specific MHCK7 promoter. Two vectors were generated in this study: MyoAAV 2A–MHCK7–mini utrophin, containing a truncated codon-optimized utrophin sequence, and MyoAAV 2A–MHCK7–YAP, containing the coding sequence of YAP. Recombinant viral particles were produced by standard triple transfection in producer cells using the AAV transfer plasmid, the plasmid encoding the MyoAAV 2A capsid, and adenoviral helper plasmids, followed by purification and titration prior to in vitro use. The AAV constructs encoded a human mini utrophin sequence and a mouse YAP sequence.

### 4.2. Animals

The mdx mouse is the most widely used animal model for DMD research, but it is important to recognize its inherent limitations. Relative to human DMD patients and large animal DMD models, mdx mice display a milder disease course and no substantial reduction in lifespan. Even so, the model recapitulates the core pathological features of human DMD with high fidelity, including skeletal muscle fiber necrosis, inflammatory cell infiltration, and the development of centrally nucleated myofibers—hallmark pathological findings of the disorder. Mdx mice also exhibit significant impairments in muscle function, most notably motor deficits such as reduced exercise endurance. These phenotypic traits align closely with the clinical manifestations of human DMD, making the mdx mouse an invaluable model for contemporary DMD research.

**Figure 5:**
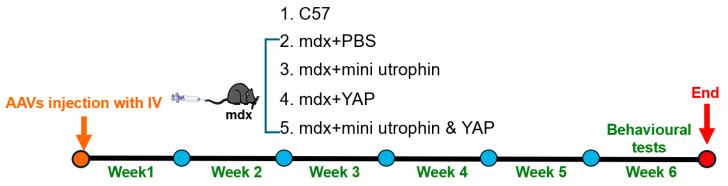
Schematic description of the study protocols in mdx. Animals were randomly assigned to four groups: C57 (wild-type control), mdx (DMD model), mdx + mini utrophin (mini utrophin treatment), mdx + YAP (YAP treatment), and mdx + mini utrophin & YAP (mini utrophin combined with YAP overexpression), with six mice per group. Behavioral assessments were performed at the end of the 5th week, followed by euthanasia and tissue harvesting at the end of the 6th week to complete the experimental protocol.

The experimental subjects comprised male C57BL/10ScSn mice and C57BL/10ScSn-Dmdmdx/J mice (mdx, purchased from Jackson Laboratory), aged 3–4 weeks. All animal experiments were approved by the Ethics Committee of the School of Sport Science, Beijing Sport University (Approval No. 2022029A). rAAVs were administered via tail vein injection: The Myo2A-MHCK7-mini utrophin was administered at a dose of 2 × 10^13^ vg/kg, and the Myo2A-MHCK7-YAP at 1 × 10^13^ vg/kg. The mdx group was administered an equivalent volume of PBS as the control group. The mice were randomly divided into five groups, with six mice per group. The following groups were used for the study: C57 (wild-type control), mdx (DMD model), mdx + mini utrophin, mdx + YAP, and mdx + mini utrophin & Yap. Behavioural testing was conducted at the conclusion of the fifth week, and mice were euthanised for the purpose of tissue collection at the termination of the sixth week, thereby finalising the experimental protocol (Figure 5).

### 4.3. Limb Grasp Strength Test

Body weight was measured before grip strength assessment. Forelimb grip strength was measured using a YLS-13A grip strength meter. Each mouse was tested three consecutive times, and the mean value was recorded as the absolute grip strength (g). To minimize inter-animal variation due to body size, relative grip strength was calculated as: Relative grip strength = absolute grip strength (g)/body weight (g). The averaged relative grip strength value for each mouse was used for statistical analysis.

### 4.4. Pole Climbing Test

A cylindrical rough wooden pole (90 cm in length, 1 cm in diameter) was placed vertically in the cage for the pole test. Mice underwent habituation training (5–10 trials) one day before testing. For each trial, mice were positioned head-up at the top of the pole, and the time to turn downward and the total time to descend were recorded. Each mouse was tested three times, and mean values were used for analysis.

### 4.5. Maximal Treadmill Exercise Test

Mice ran on a treadmill at a fixed 10% grade. Following a 5-min adaptation at 10 m/min, the speed was elevated by 3 m/min every 3 min. Exhaustion was defined as the inability to keep pace with the belt despite stimuli, at which point the total exercise time was recorded [20]. The total running time to exhaustion was recorded for each mouse and used as the indicator of endurance capacity.

### 4.6. Animal Dissection and Blood Collection

All animal experiments were performed in strict accordance with the Guidelines for the Care and Use of Laboratory Animals of the National Institutes of Health (NIH) and were approved by the Animal Ethics Committee of Beijing Sport University (Approval No. 2022029A). Every effort was made to minimize animal suffering and reduce the number of animals used.

Prior to sample collection, mice were placed in an airtight anesthesia induction chamber, and 3–5% isoflurane (mixed with oxygen at a flow rate of 1–2 L/min) was delivered to induce general anesthesia. The anesthetic depth was monitored by observing the absence of corneal reflex and paw withdrawal reflex to ensure the mice were fully anesthetized and insensible to pain. Once complete anesthesia was confirmed, blood samples were immediately collected from the tail vein. Following blood collection, mice were euthanized by cervical dislocation—a rapid and humane method that minimizes unnecessary distress, consistent with standard euthanasia guidelines for laboratory animals. Immediately after euthanasia, anatomical dissection was performed to harvest target tissues for subsequent experimental analyses.

Serum creatine kinase (CK) activity was measured using a commercial assay kit (Solarbio, #BC1145, Beijing, China) strictly following the manufacturer’s instructions.

### 4.7. Western Blotting

Following the experimental period, skeletal muscle tissues were snap-frozen in liquid nitrogen. Total protein was extracted using RIPA buffer and quantified via the BCA assay. Proteins were resolved by SDS-PAGE, transferred to membranes, and stained with No-Stain™ Protein Labeling Reagent (Thermo Fisher Scientific, Waltham, MA, USA, #A44449) for total protein normalization. Membranes were blocked and then probed overnight at 4 °C with primary antibodies against utrophin (1:300, Santa Cruz Biotechnology, Dallas, TX, USA, #sc-33699), YAP (1:1000, Proteintech, Wuhan, China, #66900-1-Ig), p-YAP (1:1000, Cell Signaling Technology, Danvers, MA, USA, #13008), GAPDH (1:8000, Transgen, Beijing, China, #HC301,), α-sarcoglycan (1:1000, Abcam, Cabridge, MA, USA, #ab189254), and γ-sarcoglycan (1:1000, Abcam, Cabridge, MA, USA, #ab203113,). After washing, membranes were incubated with IRDye^®^ 800CW Goat anti-Rabbit IgG (1:5000, LI-COR, Lincoln, NE, USA, #926-32211) or IRDye^®^ 680RD Goat anti-Mouse IgG (1:5000, LI-COR, Lincoln, NE, USA, #926-68070) for 1 h at room temperature. Fluorescent signals were detected using an LI-COR Odyssey imaging system. Band intensities were analyzed using ImageJ software (version 1.54p) and normalized to total protein stain to determine relative protein expression levels. Western blotting analyses were performed using three independent biological replicates per group. Quantification was based on biologically independent samples rather than technical replicates.

### 4.8. Hematoxylin-Eosin Staining

At the end of the experimental period, mouse skeletal muscle tissues (Triceps, Diaphragm) were fixed in neutral buffered formalin and processed into 5–8 μm paraffin sections. Hematoxylin and eosin (HE) staining was performed using hematoxylin (Solarbio, Beijing, China, #G1140), 1% acid alcohol differentiator (Solarbio, Beijing, China, #G1861), bluing reagent (Solarbio, Beijing, China, #G1866), and eosin (Solarbio, Beijing, China, #G1100), followed by dehydration in a graded ethanol series. Sections were mounted with neutral resin and imaged under a light microscope.

For quantification, at least 3 non-overlapping fields per section and at least 3 sections per muscle sample were analyzed. A myofiber was defined as centrally nucleated when one or more nuclei were clearly located within the interior of the myofiber rather than at the peripheral subsarcolemmal position. The number of centrally nucleated fibers (CNFs) and the total number of myofibers were counted manually in ImageJ/Fiji from H&E images. The percentage of centrally nucleated fibers was calculated for each field using the following formula: CNF (%) = (number of centrally nucleated fibers/total number of myofibers counted) × 100.

### 4.9. Data Analysis

HE-stained images and protein bands were analyzed using ImageJ. Data were organized with Microsoft Excel, while one-way analysis of variance (ANOVA) and data visualization were performed using GraphPad Prism 10.0.

## 5. Conclusions

The findings of this study support the therapeutic potential of mini utrophin for DMD gene therapy. In addition, our data suggest that perturbation of YAP-related signaling may influence the therapeutic response to mini utrophin in dystrophic muscle. However, because YAP activity was not directly assessed through subcellular localization or downstream transcriptional analyses, this association should be interpreted as descriptive and correlative rather than mechanistically definitive. Future studies should investigate how utrophin restoration, YAP-related signaling, and DGC-associated mechanotransductive remodeling interact in dystrophic muscle, which may help refine therapeutic strategies for DMD.

## Data Availability

All data needed to evaluate the conclusions in the paper have been provided in this article.

## Abbreviations

The following abbreviations are used in this manuscript:

DMD	Duchenne muscular dystrophy
rAAV	recombinant adeno-associated virus
YAP	Yes-associated protein
p-YAP	phosphorylated Yes-associated protein
DGC	dystrophin-glycoprotein complex
CK	creatine kinase
αSG	α-sarcoglycan
γSG	γ-sarcoglycan

## References

[1] Duan D., Goemans N., Takeda S., Mercuri E., Aartsma-Rus A. (2021). Duchenne muscular dystrophy. Nat. Rev. Dis. Primers.

[2] Dubinin M.V., Belosludtsev K.N. (2023). Ion Channels of the Sarcolemma and Intracellular Organelles in Duchenne Muscular Dystrophy: A Role in the Dysregulation of Ion Homeostasis and a Possible Target for Therapy. Int. J. Mol. Sci..

[3] Hardee J.P., Caldow M.K., Chan A.S.M., Plenderleith S.K., Trieu J., Koopman R., Lynch G.S. (2021). Dystrophin deficiency disrupts muscle clock expression and mitochondrial quality control in mdx mice. Am. J. Physiol. Cell Physiol..

[4] Song Y., Morales L., Malik A.S., Mead A.F., Greer C.D., Mitchell M.A., Petrov M.T., Su L.T., Choi M.E., Rosenblum S.T. (2019). Non-immunogenic utrophin gene therapy for the treatment of muscular dystrophy animal models. Nat. Med..

[5] Szwec S., Kaplucha Z., Chamberlain J.S., Konieczny P. (2024). Dystrophin- and Utrophin-Based Therapeutic Approaches for Treatment of Duchenne Muscular Dystrophy: A Comparative Review. BioDrugs.

[6] Fischer M., Rikeit P., Knaus P., Coirault C. (2016). YAP-Mediated Mechanotransduction in Skeletal Muscle. Front. Physiol..

[7] Aragona M., Panciera T., Manfrin A., Giulitti S., Michielin F., Elvassore N., Dupont S., Piccolo S. (2013). A mechanical checkpoint controls multicellular growth through YAP/TAZ regulation by actin-processing factors. Cell.

[8] Dupont S., Morsut L., Aragona M., Enzo E., Giulitti S., Cordenonsi M., Zanconato F., Le Digabel J., Forcato M., Bicciato S. (2011). Role of YAP/TAZ in mechanotransduction. Nature.

[9] Watt K.I., Turner B.J., Hagg A., Zhang X., Davey J.R., Qian H., Beyer C., Winbanks C.E., Harvey K.F., Gregorevic P. (2015). The Hippo pathway effector YAP is a critical regulator of skeletal muscle fibre size. Nat. Commun..

[10] Morikawa Y., Heallen T., Leach J., Xiao Y., Martin J.F. (2017). Dystrophin-glycoprotein complex sequesters Yap to inhibit cardiomyocyte proliferation. Nature.

[11] Hoffman E.P., Brown R.H., Kunkel L.M. (1987). Dystrophin: The protein product of the Duchenne muscular dystrophy locus. Cell.

[12] Campbell K.P., Kahl S.D. (1989). Association of dystrophin and an integral membrane glycoprotein. Nature.

[13] Falcucci L., Dooley C.M., Adamoski D., Juan T., Martinez J., Georgieva A.M., Mamchaoui K., Cirzi C., Stainier D.Y.R. (2025). Transcriptional adaptation upregulates utrophin in Duchenne muscular dystrophy. Nature.

[14] Wu R., Li P., Xiao P., Zhang S., Wang X., Liu J., Sun W., Chang Y., Ai X., Chen L. (2025). Activation of endogenous full-length utrophin by MyoAAV-UA as a therapeutic approach for Duchenne muscular dystrophy. Nat. Commun..

[15] Piccolo S., Dupont S., Cordenonsi M. (2014). The Biology of Yap/Taz: Hippo Signaling and Beyond. Physiol. Rev..

[16] Judson R.N., Tremblay A.M., Knopp P., White R.B., Urcia R., De Bari C., Zammit P.S., Camargo F.D., Wackerhage H. (2012). The Hippo pathway member Yap plays a key role in influencing fate decisions in muscle satellite cells. J. Cell Sci..

[17] Zhao B., Ye X., Yu J., Li L., Li W., Li S., Yu J., Lin J.D., Wang C.Y., Chinnaiyan A.M. (2008). TEAD mediates YAP-dependent gene induction and growth control. Genes Dev..

[18] Morales M.G., Gutierrez J., Cabello-Verrugio C., Cabrera D., Lipson K.E., Goldschmeding R., Brandan E. (2013). Reducing CTGF/CCN2 slows down mdx muscle dystrophy and improves cell therapy. Hum. Mol. Genet..

[19] Tabebordbar M., Lagerborg K.A., Stanton A., King E.M., Ye S., Tellez L., Krunnfusz A., Tavakoli S., Widrick J.J., Messemer K.A. (2021). Directed evolution of a family of AAV capsid variants enabling potent muscle-directed gene delivery across species. Cell.

[20] Zheng J., Lou J., Li Y., Qian P., He W., Hao Y., Xue T., Li Y., Song Y.H. (2022). Satellite cell-specific deletion of Cipc alleviates myopathy in mdx mice. Cell Rep..

